# Low Density Lipoprotein Receptor Related Proteins as Regulators of Neural Stem and Progenitor Cell Function

**DOI:** 10.1155/2016/2108495

**Published:** 2016-02-02

**Authors:** Loic Auderset, Lila M. Landowski, Lisa Foa, Kaylene M. Young

**Affiliations:** ^1^Menzies Institute for Medical Research, University of Tasmania, Hobart, TAS 7000, Australia; ^2^The School of Medicine, University of Tasmania, Hobart, TAS 7000, Australia

## Abstract

The central nervous system (CNS) is a highly organised structure. Many signalling systems work in concert to ensure that neural stem cells are appropriately directed to generate progenitor cells, which in turn mature into functional cell types including projection neurons, interneurons, astrocytes, and oligodendrocytes. Herein we explore the role of the low density lipoprotein (LDL) receptor family, in particular family members LRP1 and LRP2, in regulating the behaviour of neural stem and progenitor cells during development and adulthood. The ability of LRP1 and LRP2 to bind a diverse and extensive range of ligands, regulate ligand endocytosis, recruit nonreceptor tyrosine kinases for direct signal transduction and signal in conjunction with other receptors, enables them to modulate many crucial neural cell functions.

## 1. Low Density Lipoprotein Receptor Related Proteins 1 and 2

The LDL receptor family is a large family of multiligand receptors. Core family members include the LDL receptor; very low density lipoprotein (VLDL) receptor [1]; LDL receptor related protein (LRP)1, also known as CD91 and the *α*-2-macroglobulin receptor [2–4]; LRP2, also known as GP330 and Megalin [5]; LRP5 [6]; LRP6 [7]; and LRP8, also known as the apolipoprotein receptor-2 [8]. Each family member is a single-pass transmembrane receptor, containing two or more extracellular cysteine-rich complement type repeats, which act as ligand binding domains [9].

**Figure 1:**
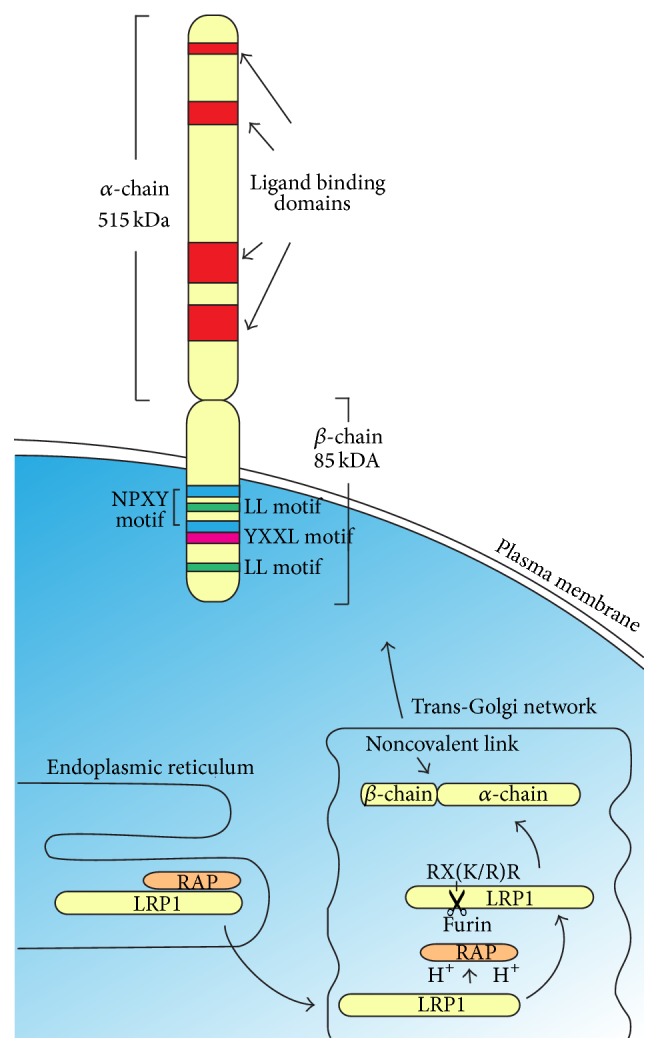
LRP1 maturation and structure. This schematic depicts the LRP1 precursor protein, which is synthesized in the endoplasmic reticulum and is bound to the chaperone protein, receptor associated protein (RAP). The LRP1 precursor is transported to the trans-Golgi network where the low pH causes RAP to dissociate. The protease Furin cleaves the LRP1 precursor at the RX(K/R)R consensus sequence to generate a large *α*-chain (515 kDa) and a smaller *β*-chain (85 kDa) which are noncovalently linked and shuttled to the cell membrane, where they are embedded as one functional unit. The *α*-chain contains four ligand-binding domains (red) that interact with a large number of ligands. The *β*-chain contains a small extracellular region, a transmembrane region which anchors the LRP1 protein within the plasma membrane, as well as two dileucine (LL, green) motifs and two asparagine-proline-x-tyrosine (NPXY, blue) motifs, where the distal motif is contiguous with a tyrosine-x-x-leucine (YXXL, pink) motif which interact with intracellular adaptor proteins and the endocytotic machinery.

At 600 kDa, LRP1 and LRP2 are the largest and most promiscuous members of the LDL receptor family. Transcription of the* Lrp1* gene can be activated by a number of transcription factors including sterol regulatory element binding protein 2 [10], hypoxia-induced factor 1*α* [11], and nitric oxide-dependent transcription factors [12], but is negatively regulated by naturally occurring antisense transcripts that are inversely coded within exons 5 and 6 of the* Lrp1* gene [13]. The* Lrp1* gene codes for a precursor protein that binds to the receptor associated protein (RAP), a chaperone that occupies the ligand binding domains of the precursor [14] to prevent the binding of other ligands [15], and ensure its correct folding in the endoplasmic reticulum [16, 17] (Figure 1). RAP remains bound to the LRP1 precursor and transports it to the Golgi apparatus. This transport involves the proximal NPXY motif in the intracellular domain of the protein [18]. In the trans-Golgi network, the low pH of the secretory pathway causes protonation of the histidine residues in domain 3 of RAP [19], triggering its dissociation from the LRP1 precursor [14, 20]. The protease Furin then cleaves the LRP1 precursor at the RX(K/R)R consensus sequence, to generate a large *α*-chain (515 kDa) and a smaller *β*-chain (85 kDa) [21]. The two fragments remain noncovalently linked on their way to the cell membrane, where they are embedded as one functional unit, comprising mature LRP1 (Figure 1). LRP2 is similarly chaperoned by RAP [22] and also contains an RX(K/R)R consensus sequence, but there is no evidence that LRP2 undergoes intracellular proteolytic processing prior to its insertion into the plasma membrane [5].

### 1.1. Soluble LRP1 and LRP2

**Figure 2:**
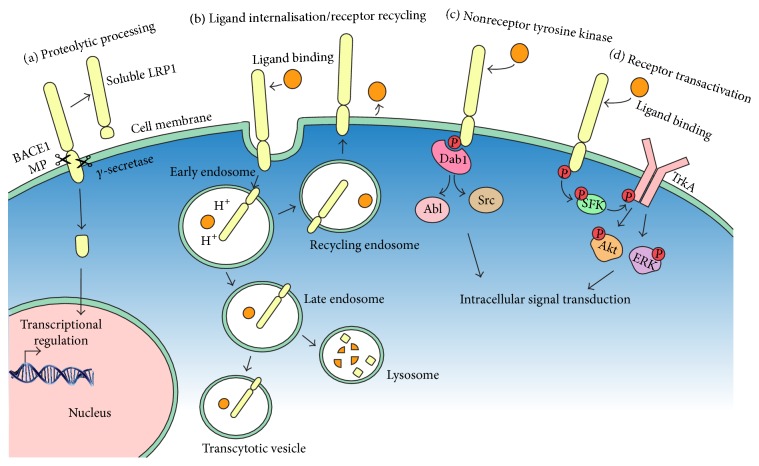
Signalling mechanisms employed by LRP1. (a) The extracellular domain of LRP1 can be shed following cleavage by beta-site APP cleaving enzyme 1 (BACE1) and metalloproteinases (MP) producing a soluble form of LRP1 (sLRP1). The intracellular domain can be cleaved by *γ*-secretase and is thought to translocate to the nucleus to influence gene transcription. (b) Ligand binding to LRP1 can result in receptor and ligand internalisation. Once internalised, the ligand/receptor complex can be processed in a multitude of ways, including degradation by lysosomes or resecretion via transcytotic and recycling vesicles. Note that while they are depicted together, ligand and receptor/s are trafficked independently. (c) Specific regions on the intracellular region of LRP1 interact with adaptor proteins such as Disabled-1 (Dab1), which interacts with the NPXY motifs and can recruit nonreceptor tyrosine kinases such as Src and Abl allowing signal transduction. (d) Activation of LRP1 by specific ligands can transactivate other receptors such as tropomyosin receptor kinase A (TrkA), which can then activate downstream signalling pathways to regulate cell function.

Once LRP1 is inserted into the plasma membrane, the soluble extracellular domain (sLRP1) can be cleaved from the cell surface by enzymes such as the beta-site APP cleaving enzyme 1 (BACE1) [23] and metalloproteinase [24] (Figure 2). sLRP1 contains the *α*-chain and a 55 kDa fragment of the *β*-chain [25] and can be detected in plasma and cerebral spinal fluid [26, 27]. Similarly, soluble fragments of LRP2 have been shown to be released from cultured choroid plexus epithelial cells and can be detected in cerebral spinal fluid [28]. LRP1 and LRP2 can also undergo intramembrane proteolysis mediated by *γ*-secretase, in either the plasma or endosomal membrane [29], to liberate an intracellular fragment which reportedly enters the nucleus [30, 31] (Figure 2(a)). The physiological function of soluble LRP fragments in normal neural cell development is poorly understood, but they have the potential to bind LRP ligands and prevent them from binding to full-length LRPs or, in the case of the intracellular domain, modulate gene transcription.

### 1.2. LRP1 and LRP2 as Mediators of Endocytosis

While the proteolytic processing of these receptors is becoming increasingly well understood, LRP1 and LRP2 remain best known for their role in mediating endocytosis (Figure 2(b)). Following ligand binding to mature LRP1 in the plasma membrane, it was originally believed that the two NPXY motifs of the cytoplasmic domain interacted with the endocytotic machinery to mediate rapid clathrin-dependant endocytosis of the receptor-ligand complex, as has been previously shown for other members of this receptor family [32]. However for LRP1, the YXXL motif and the distal dileucine motif independently mediate endocytosis, and the NPXY motifs are not required [33]. The rate of endocytosis is regulated by cAMP-dependent protein kinase A, which constitutively phosphorylates LRP1, predominantly at serine 76 of the cytoplasmic tail [34].

Like LRP1, LRP2 has two intracellular NPXY domains [5]; however unlike LRP1, the distal NPXY motif of LRP2 has been shown to interact with the phosphotyrosine-binding domain of Disabled-2 [35], a clathrin-associated sorting protein, to mediate endocytosis [29, 36, 37]. Interestingly, endocytosis does not occur during mitosis, due to the phosphorylation of Disabled-2, which removes it from the cell surface, so that it no longer colocalizes with clathrin and cannot mediate this process [38]. LRP2-directed endocytosis may still occur via clathrin-independent pathways, instead relying on the small GTPase Arf6 and caveolin 1 [39, 40]. Furthermore, LRP1- and LRP2-mediated endocytosis can be influenced by the expression of miR199a and miR199b family members, which regulate the expression of a number of genes critical for clathrin-dependent and clathrin-independent endocytosis [41]. Following endocytosis, the extracellular beta-propeller regions of LRP1 and LRP2 facilitate ligand dissociation [42], so that the ligands and receptors can be differentially sorted in early endosomes.

The mechanisms regulating the recycling of LRP1 back to the plasma membrane are not fully characterised and may vary between cell types. However, it is known that this process requires binding of the adaptor protein sorting nexin 17 to the first NPXY domain of LRP1 in early endosomes [43, 44], so that LRP1 is recycled back to the cell surface in approximately 30 minutes [45]. In early endosomes, the first NPXY domain of LRP2 instead binds the phosphotyrosine-binding domain of autosomal recessive hypercholesterolemia (ARH) [46], a clathrin-associated sorting protein that couples LRP2 to the dynein motor complex [47] and transports it from the sorting endosomes to the endocytic recycling compartment [29]. The constitutive phosphorylation of LRP2 by GSK3*β* is also involved in directing LRP2 to the endocytic recycling compartment, from which it is slowly recycled to the plasma membrane [48].

But what happens to the internalised ligand? LRP1 and LRP2 have been shown to bind upwards of 40 different ligands, many of which are structurally and functionally unrelated, and the list is always evolving [49]. They both have four LDL receptor homology regions which are the extracellular ligand-binding domains [50, 51] and bind common ligands including tissue-type plasminogen activator [52–55], apolipoprotein E, lactoferrin [17, 52], and metallothioneins I and II [56]; however not all ligands have been shown to bind both receptors. *α*2-Macroglobulin is a high affinity ligand for LRP1 [57, 58], and like prion protein has only been demonstrated to bind to LRP1 [59], while transthyretin [60] and the complex of vitamin D with the vitamin D binding protein have only been shown to bind LRP2 [61]. Once endocytosed, ligands may be degraded in lysosomes, resecreted from recycling endosomes, or trafficked in transcytotic vesicles from the apical to the basolateral membrane (or vice versa) before being secreted [62] (Figure 2(b)).

### 1.3. LRP1 and LRP2 Intracellular Signal Transduction

The true complexity of LRP1 and LRP2 signalling lies in the fact that these receptors not only trigger endocytosis but also influence signal transduction. Upon ligand binding, the NPXY motifs can function as a docking sites for intracellular adaptor proteins. LRP1 can bind cytosolic ligands in a phosphorylation-dependent manner, via two dileucine motifs and one YXXL motif in the intracellular domain. For example, the adaptor proteins Disabled-1 and FE65 can bind to the NPXY motifs of LRP1, to recruit and activate nonreceptor tyrosine kinases such as Src and Abl [63] (Figure 2(c)), allowing the receptor to transduce an intracellular signal or form signalling hubs through the binding of coreceptors [49] (Figure 2(d)). A number of coreceptors of LRP1 have been identified, including platelet-derived growth factor receptor (PDGFR) *β* [64, 65], tropomyosin-related kinase receptor A [66], amyloid precursor protein [67], and insulin-like growth factor 1 receptor [68]. These associations increase the number of intracellular pathways by which distinct LRP ligands may elicit their effects.

## 2. LRPs as Regulators of Nervous System Development

Despite the large number of common ligands and the structural similarities that exist between LRP1 and LRP2, the two genes are not functionally redundant during development. Both* Lrp1* and* Lrp2* single knockout mice have severe developmental phenotypes.* Lrp1* knockout blastocysts fail to implant and therefore do not develop into embryos [69].* Lrp2* knockout mice are mostly embryonic lethal, presenting with defects including a cleft palate, failure to form an olfactory bulb, and fusion of the forebrain hemispheres, resulting in a single ventricle (holoprosencephaly) [70]. The small number of* Lrp2* knockout mice that survive until birth experience severe vitamin D3 deficiency, as the reabsorption of vitamin D and the vitamin D binding protein from the kidney proximal tubule is LRP2-dependant, but die of respiratory failure [61, 70]. Human mutations in* Lrp2* are known to cause facio-oculo-acoustico-renal syndrome/Donnai-Barrow syndrome, an autosomal recessive disorder associated with disrupted brain formation, including agenesis of the corpus callosum [71].

The very early developmental defect observed in the* Lrp1* knockout mouse, and the gross neural phenotype of the* Lrp2* knockout mouse, do not allow us to investigate the importance of these receptors for the functioning of individual neural cell types. However, a variety of expression studies performed alongside knockdown and conditional knockout approaches demonstrate that both receptors mediate ligand endocytosis and intracellular signalling in a number of immature neural cell types. LRP1 is more widely expressed in the CNS than LRP2, being detected in mature neurons, particularly those of the entorhinal cortex, hippocampus [72] and cerebellum [73], and all CNS glia [74]. In contrast, LRP2 expression is restricted to the apical surface of the neural tube and subsequently to the forebrain, optic stalk, and otic vesicle during development [75, 76]. In the CNS of adult mice, LRP2 is predominantly expressed by cells of the choroid plexus [77] and ependymal cells [78] but has also been detected in oligodendrocytes of the spinal cord [79]. The expression patterns of LRP1 and LRP2 are largely spatially and temporally distinct, reflecting their different roles in CNS regulation.

## 3. LRP1 and LRP2 as Regulators of Neural Stem Cell Function

### 3.1. Neural Stem Cells in the Developing and Adult CNS

The early neural tube is a pseudostratified epithelium composed of neuroepithelial precursor cells. These early neural stem cells divide symmetrically, expanding their population, before switching to include asymmetric divisions that generate neuroblasts. This switch coincides with a change in gene expression, as the neuroepithelial precursor cells transition into radial glial stem cells, which comprise two molecularly distinct subgroups in the developing human brain, corresponding to those in the outer subventricular zone and those in the ventricular zone [80]. Following neuroblast generation, radial glia switch to glial generation starting with the production of oligodendrocyte progenitor cells (OPCs) and concluding with the production of astrocytic precursors [81]. Towards the end of development a subset of radial glial stem cells adopt a more astrocytic gene expression profile and give rise to the adult neural stem cells [82].

In adulthood neural stem cells reside in two key niches, the subventricular zone of the lateral ventricles and the dentate gyrus of the hippocampus, where they proliferate to generate intermediate progenitor cells and ultimately neuroblasts [83]. Neural stem cells in the subventricular zone also produce a small number of OPCs under normal physiological conditions [84]. The behaviour of neural stem cells (and their intermediate progenitors) is highly controlled by mitogenic and morphogenic signalling. While key ligands and receptors for these pathways are well described, the role of LRP1 and LRP2 in these pathways has only recently been elucidated.

### 3.2. LRPs as Regulators of Cell Fate Specification

LRP1 and LRP2 have both been shown to facilitate the internalisation of the potent morphogen, sonic hedgehog [85–87], a finding that has provided insight into the significant neurodevelopmental defects observed in patients and mice lacking normal functioning* Lrp2* [70, 71, 75]. LRP2 is expressed by neuroepithelial cells, on the apical side of the neural plate, as early as E7.5 in the mouse. After neural tube closure at E9.5, LRP2 expression becomes increasingly restricted to the midline, ultimately being localized to the clathrin-coated pit regions of the apical cell membrane, clustered at the base of the primary cilium (a cellular organelle essential to sonic hedgehog signalling) [88] and in the subapical endosomes of the radial glia [89]. At E8 sonic hedgehog is produced by cells of the axial mesoderm (the notochord and prechordal plate) and by E8.5 its expression expands to include the radial glia at the ventral midline of the rostral diencephalon. This expansion does not occur in* Lrp2* knockout embryos, as LRP2 is required for the radial glia to bind and sequester sonic hedgehog as it diffuses, regulating morphogen presentation to the neural stem cells [89].

Once sonic hedgehog is bound to LRP2 it can also bind its receptor patched-1, and the complex undergoes clathrin-mediated endocytosis [89]. All components can then be found within early endosomes and recycling endosomes but do not appear to be targeted to the lysosome for degradation. The internalisation of patched-1 by LRP2 results in activation of the effector smoothened, leading to changes in gene transcription mediated by the Gli transcription factors. Therefore, in the absence of LRP2, radial glia show reduced expression of the sonic hedgehog target genes* gli1* and* six3* [89]. The loss of sonic hedgehog and Gli3-mediated transcriptional repression has secondary consequences for neural development, including aberrant bone morphogenic protein 4 expression in the dorsal forebrain [75, 89, 90] and disrupted fibroblast growth factor 8 and noggin expression [89]. These data indicate that LRP2 regulates the patched-1-dependent internalisation and trafficking of sonic hedgehog [89], which is necessary for neural stem cell specification and ventral forebrain patterning.

Later in development, the expression of LRP2 by spinal cord radial glial is also necessary for glial cell specification.* Lrp2* knockout mice completely lack oligodendrocyte-lineage cells and produce very few astrocytes in the spinal cord [91]. OPC specification from radial glia in the ventral spinal cord is also directed by sonic hedgehog signalling [92–96], and so the lack of spinal cord oligodendrocytes may be explained by a mechanism similar to that detailed above. However OPCs can be generated from cultured neuroepithelial precursors derived from* sonic hedgehog* and* smoothened* knockout mice [97, 98], indicating that LRP2 must also interact with other signalling pathways such as basic fibroblast growth factor and insulin-like growth factor I [99], to promote OPC generation from neural stem cells. The decreased number of astrocytes observed in* Lrp2* knockout mice is also interesting. LRP2 is expressed by vimentin-positive cells in the E15 ventral spinal cord [79] that most likely correspond to immature astrocytes [84, 100, 101]. While LRP2 may play a role in regulating the behaviour of astrocytic precursors, it is more likely that the observed phenotype is the result of LRP2 being required for astrocyte specification by radial glia, as this immature glial population is not generated in* Lrp2* knockout mice. Despite these observations that strongly implicate LRP2 in glial cell specification during neural development, the ligands and signalling mechanisms are unknown.

LRP1 appears to fulfill a similar role in regulating glial generation in the brain. LRP1 is expressed by cells within the embryonic ventricular zone and the early postnatal subventricular zone [102]. While the role of LRP1 in regulating neural stem cell function* in vivo* is poorly understood,* in vitro* studies suggest that LRP1 can regulate OPC production. Neural stem cells can be harvested from the cortex of embryonic mice and grown as a suspension culture termed neurospheres. When differentiated, neurospheres generate neurons, astrocytes, and oligodendrocytes. However, neurospheres lacking* Lrp1* generate normal numbers of neurons, but significantly fewer O4-positive oligodendrocytes [102]. These data may reflect a requirement of LRP1 signalling in neural stem cells for OPC specification but could equally result if LRP1 is necessary for the proliferation or differentiation of OPCs (see OPC section below).

### 3.3. LRPs as Regulators of Neural Stem Cell Proliferation

In the subventricular zone of the adult mouse brain, LRP2 is expressed by ependymal cells underlying the neurogenic niche [78, 103]. The importance of LRP2 expression for neural stem and progenitor cell proliferation was examined in* Lrp2* ^*267/267*^ mutant mice, which produce a truncated form of LRP2 [104].* Lrp2* ^*267/267*^ mice have ~25% fewer proliferating cells in the subventricular zone relative to control mice and a proportional reduction in the number of newborn neurons entering the olfactory bulb [78]. The absence of functional LRP2 from the neurogenic niche was accompanied by increased bone morphogenic proteins 2 and 4, increased phosphorylation of the downstream effectors SMAD1, SMAD5, and SMAD8, and increased activation of the downstream target, inhibitor of DNA binding 3 [78]. It is known that LRP2 can act as an endocytic receptor, sequestering and clearing bone morphogenic protein 4 [75]. However this does not appear to be the mechanism at play here. A ventricular infusion of noggin, the potent bone morphogenic protein 4 antagonist [105], certainly decreases neurogenesis but does so in favour of oligodendrogenesis [106], and this fate-switch is not consistent with the phenotype of the* Lrp2* ^*267/267*^ mouse [78].

The ability of LRPs to regulate proliferation may be more widespread amongst immature neural cell populations, as LRP1 also regulates the proliferation of cerebellar granular neuron precursors. Cerebellar granular neuron precursors are a temporary cell population that proliferate in the external germinal zone of the developing cerebellum, producing granule neurons from birth until ~P15 in the mouse [107]. This cell population is highly responsive to the promitotic effects of sonic hedgehog [108–110]. However, the effect of sonic hedgehog is negatively regulated by an interaction between LRP1 and protease nexin 1, also known as SERPINE2. Protease nexin 1 complexes with its target proteases and binds to LRP1 on the surface of cultured cerebellar granule neuron precursors [111]. Once endocytosed, protease nexin 1 antagonizes sonic hedgehog signalling, reducing the proliferation of cerebellar granule neurons. This regulation is critical for normal cerebellar development, as the absence of protease nexin 1* in vivo* delays cerebellar granule neuron precursor differentiation and increases the overall size of the cerebellum [111]. We would predict that conditionally removing* Lrp1* from cerebellar granule neuron precursors would have the same effect.

## 4. LRPs as Regulators of Neuroblast Function

Neuroblast generation and their subsequent migration into the developing cortex has been well characterised [112]. Postmitotic neuroblasts that are generated in the cortical ventricular zone are destined to form cortical projection neurons [113]. They undergo radial migration out of the germinal zone, moving along the apical processes of radial glia. The final laminar position of a newborn neuron is determined by its birth date, with late-born neuroblasts migrating past early-born neurons, to seed progressively more superficial layers of the cortex [114]. In contrast, cortical interneurons are generated from radial glial cells within the ventricular zones of the medial ganglionic eminence, the caudal ganglionic eminence and the preoptic area, and undergo both radial and tangential migration to populate each of the cortical layers [115–117].

Neuroblasts born in the two neurogenic niches of the adult brain also have vastly different migratory requirements. Those born in the hippocampus are destined to be dentate granule neurons, and send axons from the dentate gyrus to CA3 of the hippocampus [118]. After birth these cells move a very short distance as they mature, migrating from the subgranular zone (the inner lip) of the dentate granule neuron layer to their final position within the layer. On the other hand, neuroblasts born in the subventricular zone migrate tangentially, moving as neuroblast chains through the rostral migratory stream into the olfactory bulb [119]. Upon exiting the rostral migratory stream, the neuroblasts turn and migrate radially and differentiate into granule and periglomerular neurons in the olfactory bulb [83]. This type of chain migration is regulated by signals that modify the actin cytoskeleton including contact-mediated signalling between the neuroblasts and the ensheathing glia [120–123] and the chemorepulsion mediated by slit and netrin [124–126]. Recent evidence suggests that, following neural stem cell specification and neuroblast generation, LDL family members, including LRP1 and LRP2, continue to play a significant role in regulating the successful maturation and integration of these new cells in the CNS.

### 4.1. LRP8 and the VLDL Receptor Are Key Regulators of Neuroblast Migration in Development and Adulthood

While this review focuses on LRP1 and LRP2, it is not possible to discuss the role of LDL family members in regulating neuroblast migration without first detailing the importance of the LRP8 and VLDL receptor in cortical development.* Lrp8* and* VLDL receptor* double knockout mice have abnormalities in the layering of the brain, including the ectopic placement of neurons [127, 128], and also exhibit malformation of the cerebellum and spinal cord [127, 129]. LRP8 and the VLDL receptor are high affinity receptors for reelin [130, 131] a large extracellular matrix protein [127, 129, 130]. Mice that lack* reelin* largely phenocopy the distinct cortical lamination defects seen in the* Lrp8* and* VLDL receptor* double knockout mice [127, 129, 130]. Oligomeric reelin binds to LRP8 and the VLDL receptor, activates Src family kinases, and induces phosphorylation of Disabled-1. This signalling pathway enables polarisation, adhesion, stabilisation, process outgrowth, and ultimately neuroblast migration [132–134]. During development reelin is first expressed in the cortical marginal zone by Cajal-Retzius cells [135–137] and later by interneurons [138, 139]. Humans with mutations of the* VLDL receptor* gene have an increased risk of developing schizophrenia, which is thought to result from subtle neuroblast migration defects within the brain [140].

LRP8 and the VLDL receptor can also regulate neuroblast migration when activated by an alternative ligand, thrombospondin-1. Thrombospondin-1 is expressed in the subventricular zone and throughout the rostral migratory stream [141], where it acts on LRP8 and the VLDL receptor to promote neuroblast chain migration.* Thrombospondin-1* knockout mice have defective chain migration, with fewer neuroblasts successfully migrating to the olfactory bulb [141]. This phenotype is also observed in mice lacking LRP8 and VLDL receptor, or Disabled-1, but is not observed in* reelin* knockout mice [142]. However, the successful migration of neurons from the subventricular zone to the olfactory bulb appears to require both ligands. Thrombospondin-1 stabilizes neuroblast chains and increases their length in the subventricular zone and rostral migratory stream, but reelin, produced by mitral cells in the olfactory bulb, is a higher affinity ligand and subsequently directs neuroblast dissociation, allowing them to transition to radial migration [143]. Of the two ligands, only reelin activates the proteasomal degradation of Disabled-1, which is necessary for neuroblast dissociation [141].

There is no evidence that reelin signalling interacts with LRP1 or LRP2. However, thrombospondins are known to interact with membrane proteins such as integrins, CD47, CD36, proteoglycans, and LRP1. Thrombospondin-1 has been shown to interact with LRP1 in combination with calreticulin to promote the focal adhesion of mature oligodendrocytes [144] and microglia [145] but has not been demonstrated to regulate neuroblast migration.

### 4.2. LRPs, Neuroblast Migration, and Neuronal Development

LRP2 regulates neuroblast migration indirectly.* In vitro* LRP2 and caveolins are expressed by astrocytes and work together to bind and endocytose albumin [40, 146]. This is significant, as albumin uptake activates the transcription factor sterol regulatory binding element protein 1, inducing expression of stearoyl-coA 9-desaturase-1, the key enzyme required for synthesis of the neurotrophic factor oleic acid [147]. In the lateral periventricular zone of the developing rat brain, oleic acid production regulates neuronal growth, migration, axon generation, and early synaptogenesis [148, 149], with the major neurotrophic effect being mediated by the downstream effectors PAR-*α*, protein kinase A, and neuro D2 [150]. When* stearoyl-coA 9-desaturase-1* is knocked down in lateral periventricular explant cultures, albumin-mediated neuroblast migration is essentially prevented [148].

Once neuroblasts stop migrating, their journey is far from over. The immature neurons extend an axonal process to commence formation of the circuitry of the nervous system. The extending axons are tipped with a growth cone, which navigates the extracellular matrix, guiding the axon to its target cell to ultimately form a synapse [151]. A growth cone comprises membranous, receptor-rich, fan-shaped lamellipodia that extend along finger-like projections known as filopodia. The growth cone cytoskeleton is comprised of closely interacting microtubules and filamentous and globular actin [152–154]. Bundles of filamentous actin give structure to the filopodia, as does the cross-linked filamentous actin along the lamellipodial leading edge [152, 154, 155]. Microtubules are arranged as parallel bundles along the axon and splay outwards within the growth cone, providing structure and transport for proteins and organelles [156].

Growth cones are fitted with an elaborate suite of receptors that allow for the simultaneous integration of a multitude of chemotactic cues [157]. Binding of a chemotactic factor to its specific receptor/s on the growth cone membrane induces an intracellular signalling cascade which manipulates the cytoskeletal elements and dictates whether the response of the growth cone culminates in turning, extension, stasis, retraction, collapse, or bifurcation [154]. Well-defined receptors for chemotactic signals include the Eph family of receptor tyrosine kinases, Neuropilin, Roundabout, Deleted in Colorectal Cancer, L1, and Plexins (reviewed in [158]).

LRP1 and LRP2 are highly expressed on the growth cones of developing neurons* in vitro* and have been shown to signal in a codependent manner to promote chemotactic axon guidance within developmental neurons* in vitro* [159]. Together, LRP1 and LRP2 act as chemotactic receptors for a variety of ligands, including metallothioneins and tissue-type plasminogen activator [159]. Metallothioneins are small, highly conserved, inducible heavy metal binding proteins that are avid scavengers of reactive oxygen species [160]. Metallothioneins I and II are widely expressed in the nervous system and elsewhere. They differ by only a few amino acids and appear to have redundant functions. Metallothionein III is highly expressed in the brain, while metallothionein IV appears to be absent from the nervous system [161]. In cultured growth cones from sensory neurons, the activation of LRP1 and LRP2 by metallothionein II stimulated chemoattraction, resulting in growth cones turning towards the source of metallothionein II [159]. Metallothionein III had the opposite effect and induced chemorepulsion. Other LRP1 ligands, such as *α*2-macroglobulin, and tissue-type plasminogen activator also induced chemorepulsion [159]. The opposing responses induced by different LRP1 ligands are thought to result from differential activation of downstream signaling pathways, with metallothionein II activating Ca^2+^/calmodulin-dependent protein kinase and other receptors such as the tropomyosin-related kinase A receptor in complex signaling hubs (see Figure 2(d)).

Various LRP ligands have also been shown to alter neurite outgrowth. For example, metallothionein I/II signalling has been shown to transiently activate Akt and ERK, which belong to the mitogen-activated protein kinase and the phosphoinositide-3 kinase/Akt intracellular signalling pathways [162]. Myelin associated glycoprotein, an established chemorepulsive molecule, is known to interact with LRP1 [163] to inhibit axonal outgrowth and induce growth cone collapse [164, 165].* In vitro* experiments have demonstrated that myelin associated glycoprotein and LRP1 form a complex with the p75 neurotrophin receptor, to activate RhoA [163], a potent mediator of growth cone collapse and axon retraction [166]. Additionally apolipoprotein E-containing lipoproteins are secreted by astrocytes and have been shown to bind LRP1 on the surface of immature neurons to promote neurite outgrowth generally, without having an effect on directionality [167]. The complexity of LRP signaling interactions in immature neurons remains to be fully deciphered but appear to be context- and ligand-dependent [168].

Mice in which* Lrp1* is selectively deleted from neurons exhibit prominent tremor and dystonia, behavioural abnormalities, hyperactivity, motor dysfunction, age-dependent dendritic spine degeneration, synapse loss, neuroinflammation, memory loss, eventual neurodegeneration, and premature death [169–171], clearly demonstrating that LRP1 is crucial to neuronal function. LRP1 is also found postsynaptically, where it can interact with NMDA receptors* in vitro*, via the intracellular scaffold postsynaptic density protein 95 [169, 172]. LRP1 is able to influence the activity of NMDA receptors and regulate their distribution and internalisation [168, 173, 174], as well as the NMDA-induced internalisation of the AMPA receptor subunit GluR1 [174]. The very nature of this LRP1/NMDA receptor relationship suggests that LRP1 plays an integral role in neurotransmitter-induced calcium signalling, particularly in synaptic plasticity [173, 174].

LRP8 also regulates synaptic plasticity [128, 175]. LRP8 activation, by the addition of reelin to primary mouse cortical neuron cultures, triggers its proteolytic cleavage by *γ*-secretase. The liberated intracellular domain translocates to the nucleus, along with phosphorylated CREB to enhance the transcription of genes associated with learning and memory [176]. Furthermore, the ability of neurons to produce ATP for synaptic transmission may be tied to LRP1, as cultured neurons lacking* Lrp1* have reduced expression of the glutamate transporters GLUT3 and GLUT4 [177].

## 5. LRP1 and LRP2 as Regulators of Oligodendrocyte Progenitor Cell Function

OPCs, also known as NG2 glia, are a proliferative, immature cell type found in the developing and adult CNS [178, 179]. OPCs can be identified by their expression of specific proteins such as the NG2 proteoglycan [180] and PDGFR*α* [181]. During the early stages of embryonic development, OPCs are produced from radial glia in the neuroepithelium of the developing brain and spinal cord [182, 183]. In the mouse spinal cord, OPC generation commences from the ventral pMN domain at E12.5 [182, 184]. The pMN domain is named for its role in generating spinal cord motor neurons and is defined by the expression of two transcription factors, OLIG1 and OLIG2 [185], both of which are highly expressed by OPCs and necessary for their generation and subsequent differentiation [186, 187].* Olig1/2* expression by pMN domain neural stem cells is induced by a gradient of ventrally secreted sonic hedgehog, suggesting that specification of this domain would also be LRP1/2-dependant. In the absence of* Olig1/2*, stem cells in the pMN domain instead form V2 interneurons and astrocytes [188]. Shortly after their birth, OPCs differentiate into myelinating oligodendrocytes in the spinal cord grey and white matter [183, 189]. It is estimated that approximately 85% of all spinal cord oligodendrocytes originate from the pMN domain, but other domains such as the P3 domain [184] and more dorsal domains [190, 191] also produce OPCs, just slightly later in response to different spatiotemporal cues.

Like spinal cord OPCs, forebrain OPCs have multiple origins. They are generated and migrate in three distinct waves [192]. The initial wave commences in the medial ganglionic eminence and the anterior entopeduncular area at E12.5 in mice. The OPCs migrate from their ventral origins to populate all regions of the developing brain, including the developing cortex [96]. The next wave of OPCs is initiated at E15.5 from the lateral- and caudal-ganglionic eminence, followed by the third and final wave from the cortical neuroepithelium [192]. OPCs derived from the initial wave are lost shortly after birth [192] and the function performed by these temporary OPCs and the signals rendering them susceptible to developmental removal are still unknown. By P13, ~80% of oligodendrocyte-lineage cells in the corpus callosum originate from the cortical neuroepithelium, and the remainder originate from the lateral ganglionic eminence [191]. All OPCs that populate the optic nerve arise from the preoptic area [193].

LRP1 may be a critical regulator of OPC behaviour, as recent microarray [194] and RNA sequencing [195] data indicate that* Lrp1* mRNA is highly expressed by OPCs in the early postnatal mouse brain. However expression of this gene is rapidly downregulated upon differentiation and is barely detectable in oligodendrocytes. The role of LRP2 in regulating this lineage is more clearly established.

### 5.1. LRP2 Regulates OPC Proliferation and Migration during Development

One of the signalling molecules regulating OPC proliferation and migration is sonic hedgehog [196, 197], and LRP2 appears to regulate OPC proliferation and migration by modulating sonic hedgehog availability and contributing to the generation of a concentration gradient. In the developing mouse optic nerve, LRP2 is highly expressed by astrocytes [198]. However, LRP2 expression is not homogeneous, being highest in the caudal optic nerve at E14.5, but then changing to be highest in the rostral optic nerve at E16.5. Blocking LRP2 signalling by optic nerve astrocytes leads to a significant reduction in OPC proliferation and migration [198].* In vitro* studies suggest that the LRP2-mediated uptake and release of sonic hedgehog by astrocytes promotes OPC proliferation and act as a chemoattractant directing their migration [198]. The temporal regulation of LRP2 expression in the caudal versus rostral regions of the optic nerve would be predicted to “trap” sonic hedgehog in the region being populated by OPCs at that time. The expression pattern of LRP2 in the postnatal optic nerve has not been characterised. However as LRP2 is expressed by mature oligodendrocytes in the postnatal spinal cord [199], it might also be upregulated by optic nerve OPCs upon differentiation.

### 5.2. How Might LRP1 Influence OPC Behaviour?

When examining LRP1 function in other cell types, there are a number of mechanisms by which LRP1 could feasibly influence OPC behaviour. For example OPC processes share some structural similarities with the growth cones of developing neurons [200, 201]. In particular growth cones comprise specialised cell membrane extensions called lamellipodia and filopodia, which also extend from the cellular processes of OPCs [200]. LRP1 signalling mediates the chemoattraction and chemorepulsion of growth cones* in vitro*, [159], so perhaps LRP1 could regulate OPC process guidance or even OPC migration. LRP1 is expressed by Schwann cells* in vivo* and regulates the migration and adhesion of immature Schwann cells* in vitro* by the activation and repression of two small Rho GTPases, Rac1 and RhoA, respectively [202]. Rac1 activation stimulates the formation of peripheral lamellae by actin remodelling in the leading process [203].* Lrp1* knockdown decreases Rac1 activation and increases RhoA activation, which in turn increases cell adhesion and prevents migration [202]. This is of particular interest, as OPCs take on a bipolar morphology when migrating [201], and their movement has been attributed to the NG2-dependent regulation of small Rho GTPases and polarity complex proteins [204].

LRP1 also has the potential to influence OPC migration by acting as a coreceptor for PDGFR*α* signalling, in a similar way that it promotes fibroblast migration by cosignalling with PDGFR*β*. When PDGFBB binds to PDGFR*β* on the surface of cultured mouse embryonic fibroblasts, it induces migration. However this involves the association of LRP1 with PDGFR*β* [205, 206]. The two receptors are internalised and colocalize in the endosomal compartment, where the kinase domain of PDGFR*β* phosphorylates the distal NPXY motif of LRP1 [65, 205, 207]. Once phosphorylated, LRP1 has an increased affinity for the intracellular domain for SHP-2 [206, 208], outcompeting PDGFR*β* for this interaction, and preventing further activation of downstream signalling pathways [206]. While OPCs do not express PDGFR*β*, they express high levels of the related receptor, PDGFR*α*, which is also internalised following ligand binding [209], suggesting an association with an unidentified endocytic receptor which we propose could be LRP1. PDGFAA is known to bind to PDGFR*α* on the surface of OPCs and activate a phosphorylation cascade involving the Fyn tyrosine kinase and cyclin-dependant kinase 5 [210], a known regulator of the actin cytoskeleton in neurons [211]. By interacting with PDGFR*α* it is feasible that LRP1 could promote not only OPC migration but also proliferation and cell survival [181, 210, 212, 213]. While the signalling mechanism is likely to be different, a role for LRP1 in regulating cell survival is not unprecedented, as LRP1 has been shown to protect Schwann cells against TNF*α*-induced cell death in a sciatic nerve crush injury model* in vivo* and* in vitro* [214].

LRP1 could equally influence OPC migration by regulating lipid availability within the cell, as the establishment of cell polarity and movement of the leading edge during migration is dependent on the availability of cholesterol [215, 216]. Most lipid-carrying proteins cannot cross the blood brain barrier and therefore must be generated within the CNS. Apolipoprotein E is secreted by astrocytes and functions as an effective lipid transport protein and can bind LRP1 [217, 218]. Lipoproteins form noncovalent aggregates with triglycerides, phospholipids, and cholesterol esters before they bind to specific receptors and are internalised and utilized by the cell [219]. Upon binding of apolipoprotein E to LRP1, the complex is internalised where its lipid content is discharged, making it available to the cell [220], before apolipoprotein E is resecreted [221]. Once internalised, lipoproteins may be utilized by OPCs for a number of functions.

LRP1-mediated lipid uptake may alternatively allow OPCs to sustain their postsynaptic connections with neurons. Forebrain neuron-specific* Lrp1* gene knockout mice have severe deficiencies in lipid metabolism and show synapse loss [171]. The presynaptic use of cholesterol by neurons is high, due to the requirements of lipid-rich neurotransmitter vesicles [222]. However, the postsynaptic cell also utilizes cholesterol for receptor recycling in and out of the postsynaptic membrane. Therefore, cholesterol uptake into OPCs may be critical for the formation of axon-OPC synapses and maintenance of the OPC postsynaptic density.

## 6. Conclusions and Outlook

Our knowledge of LRP1 and LRP2 processing and trafficking has come a long way in the past decade. Without even considering the possibility that cleaved forms of these proteins may regulate gene transcription or perform dominant negative signalling functions, a growing number of studies clearly indicate that LRP1 and LRP2 perform a diverse range of cellular functions in neural stem and progenitor cell populations. The generation of conditional knockout mice has now made it possible to perform the detailed studies that will be necessary to understand the role of LRP1 and LRP2 in each immature cell type, across a variety of developmental stages. This is particularly critical now that we understand that LRP1 and LRP2 can influence the balance of growth factor and morphogen signalling, making them critical spatial and temporal regulators of neural development.
